# MiR-10a-5p suppresses hepatocellular carcinoma progression and microvascular invasion by targeting TFR1-STAT3-CD24 signaling axis

**DOI:** 10.3389/fonc.2025.1694441

**Published:** 2026-01-02

**Authors:** Yupeng Zhang, Di Lai, Ziyi Wang, Xiaoyue Nong, Yingying Chen, Rulin Xu, Tao Bai, Wei Zhang

**Affiliations:** 1 Department of Radiology, Liuzhou People’s Hospital, Guangxi Medical University, Liuzhou, China; 2 Clinical Science Research Department, CT & MR Business Division, Canon Medical Systems (China), Guangzhou, China; 3 Department of Hepatobiliary Surgery, Guangxi Medical University Cancer Hospital, Nanning, China

**Keywords:** hepatocellular carcinoma, CT, MiR-10a-5p, CD24, transferrin receptor 1, STAT3

## Abstract

**Background:**

Preoperative CT features have demonstrated potential in predicting microvascular invasion (MVI) in hepatocellular carcinoma. These CT signs serve as indicators of tumor biological behavior. However, the molecular mechanisms underlying the ability of CT to preoperatively predict MVI remain largely unexplored. This study aims to elucidate the role of miR-10a-5p in HCC progression and investigate its correlation with specific imaging features.

**Methods:**

According to preoperative CT features, patients were divided into CT-MVI (+) group and CT-MVI (-) group. MiR-10a-5p expression was analyzed in HCC tissues with CT-MVI compared to those without CT-MVI. The functional effects of miR-10a-5p were assessed through *in vitro* cell viability and apoptosis assays, and *in vivo* xenograft models. Cell viability was assessed through the CCK8 assay, while flow cytometry was utilized to determine cell apoptosis. The regulatory relationships between miR-10a-5p, CD24, transferrin receptor 1 (TFR1), and STAT3 signaling were explored using western blot, luciferase reporter assays, and rescue experiments.

**Results:**

MiR-10a-5p expression was significantly downregulated in HCC tissues with CT-MVI compared to those without CT-MVI. Overexpression of miR-10a-5p reduced cell viability, promoted apoptosis, and inhibited tumor growth *in vivo*. MiR-10a-5p directly targeted TFR1, leading to decreased CD24 expression and reduced STAT3 phosphorylation. TFR1 overexpression partially rescued the effects of miR-10a-5p on cell apoptosis and CD24 expression.

**Conclusions:**

Our findings reveal miR-10a-5p as a key regulator of HCC progression and MVI, operating through a novel pathway involving TFR1, STAT3, and CD24. This study provides new insights into the molecular mechanisms of CT features and highlights potential therapeutic targets for HCC treatment.

## Introduction

Microvascular invasion (MVI) is a critical prognostic factor in hepatocellular carcinoma (HCC), significantly influencing recurrence rates and overall survival following surgical resection or liver transplantation (1–3). MVI indicates an aggressive tumor behavior and is a well-known predictor of early recurrence within the first two years post-treatment (4). Our previous research results show that despite the standardized antiviral and chemotherapy after surgery for patients with positive MVI, the recurrence rate in three years after surgery still reaches 50% (5). Our researches and previous researches have demonstrated that certain imaging features on CT, can serve as biomarkers for predicting MVI or postoperative early recurrence (6–10). These features include tumor margins, tumor size, and tumor capsule (6, 7). MVI is closely linked to tumor immune escape. As cancer cells invade small blood vessels, they can evade immune surveillance more effectively. MVI provides a route for tumor cells to spread, while also creating an environment that suppresses local immune responses, ultimately facilitating tumor progression and metastasis (11).

Cancer cells employ various mechanisms to evade immune surveillance and promote tumor growth (12). Recent research has highlighted the roles of CD24 and the transferrin receptor 1 (TFR1) in facilitating tumor immune escape, as well as the importance of iron homeostasis in cancer development, particularly in HCC. CD24 has emerged as a key player in tumor immune evasion (13). This small glycoprotein interacts with Siglec-10 on tumor-associated macrophages, triggering inhibitory signaling that suppresses anti-tumor immune responses. By engaging Siglec-10, CD24 acts as a “don’t eat me” signal, preventing macrophage-mediated phagocytosis of cancer cells. This CD24-Siglec-10 axis represents a novel immune checkpoint that allows tumors to evade innate immune surveillance (14, 15).

The TFR1 contributes to tumor immune escape through its central role in cellular iron uptake (16). TFR1 is often overexpressed on cancer cells to meet their increased iron demands for rapid proliferation (17, 18). Beyond supporting tumor growth, intracellular iron accumulation has been shown to protect cancer cells from natural killer (NK) cell-mediated killing. By modulating iron levels, TfR1 helps create an immunosuppressive tumor microenvironment that impairs anti-tumor immunity (19). Iron homeostasis plays a particularly crucial role in the development and progression of liver cancer. The liver is a major site of iron storage and metabolism in the body (20). Dysregulation of iron homeostasis, often manifesting as iron overload, is strongly associated with an increased risk of HCC. Excess intracellular iron can promote oxidative stress, DNA damage, and alterations in cell signaling pathways that drive hepatocarcinogenesis (21, 22). Furthermore, iron accumulation in HCC cells appears to maintain cancer stem cell-like properties and enhance malignant behaviors (23).

In the regulation of tumor immune escape, microRNAs play a crucial role by influencing various immune response aspects. These tiny non-coding RNA molecules have the ability to alter the expression of genes related to immunity, impacting both innate and adaptive immune systems. Certain microRNAs can inhibit anti-tumor immune responses through their effects on essential immune cell functions, the production of cytokines, or the presentation of antigens (24). In contrast, some microRNAs potentially boost immune surveillance. Cancer cells frequently take advantage of this regulatory system, manipulating microRNA expression to establish an environment that suppresses immune function, thereby avoiding detection and destruction by the immune system (25). Our research utilized RNA sequencing to analyze the miRNA expression profile HCC. The results showed a significant decrease in miR-10a-5p levels within preoperative CT features indicated MVI positive. We sought to determine if this reduced expression of miR-10a-5p correlates with the mechanisms underlying CT-MVI features and the ability of HCC to evade immune responses. Our study not only sheds light on the molecular mechanisms underlying CT-MVI features in HCC but also opens up new avenues for integrating imaging features with gene and protein expression profiles. The correlation between imaging characteristics and molecular markers presents an exciting opportunity for non-invasive assessment of HCC’s genetic landscape and potential for MVI.

## Materials and methods

The experimental design, implementation, and reporting adhered to the ARRIVE guidelines (https://arriveguidelines.org). All procedures performed in this study were in accordance relevant animal guidelines and regulations. All experiments involving human participants and human-derived materials were conducted in compliance with the Declaration of Helsinki and approved by the Ethics Committee of Liuzhou People’s Hospital (KY2024-067-01) and Guangxi Medical University Cancer Hospital (LW2024116). Informed consent was obtained from all participants (or their legal guardians) prior to sample collection and analysis. Pathological MVI assessment was performed independently as part of routine workflow and was not informed by the CT-based grouping. This study was designed as a pilot radiogenomic investigation. Because the primary aim was to explore potential imaging–molecular associations and generate preliminary mechanistic hypotheses, a formal sample size calculation was not performed. The limited sample size should therefore be interpreted within the context of an early-stage exploratory study.

### Cell culture and treatment

Hep3B and Huh7 cells were obtained from the American Type Culture Collection and cultured in Dulbecco’s Modified Eagle’s Medium (DMEM; Gibco, Waltham, MA, USA) supplemented with 10% fetal bovine serum (FBS; Gibco, Waltham, MA, USA) and 1% penicillin-streptomycin. The cells were maintained at 37°C in a humidified atmosphere with 5% CO2. The miR-10a-5p mimic negative control (Mimic-NC), miR-10a-5p mimic (Mimic-10a), miR-10a-5p inhibitor negative control (Inhibitor-NC), and miR-10a-5p inhibitor (Inhibitor-10a) were purchased from Guangzhou RIBOBIO Co. Ltd. (Guangzhou, China). Desferrioxamine (DFO) was obtained from Sigma, dissolved in sterile deionized water, and used at a concentration of 100 μM. For TFR1 overexpression, the full-length CDS of TFR1 was cloned into the pcDNA3.0 vector. The sequences used for TFR1 knockdown using siRNA (Genepharma, Shanghai, China) are as follows: si-TFR-1-sense: UAAGUCAAAAGGUCAAUUCUG; si-TFR-1-antisense: GAAUUGACCUUUUGACUUAAA; si-TFR-2-sense: UAAUUGAUCACCACGAAUGGG; si-TFR-2-antisense: CAUUCGUGGUGAUCAAUUAAA; si-TFR-3-sense: AAUUUUGCAGGAGUACUACCA; si-TFR-3-antisense: GUAGUACUCCUGCAAAAUUUU; Control-sense: UUCUCCGAACGUGUCACGUTT; Control-antisense: ACGUGACACGUUCGGAGAATT. All transfections were performed using the Lipo™ 6000 transfection reagent (Beyotime Biotechnology, Shanghai, China) according to the manufacturer’s instructions.

### Quantitative real-time PCR

Total RNA and miRNA were isolated from cells or tissues using the miRNeasy Micro Kit (Qiagen, Hilden, Germany) following the manufacturer’s instructions. The PrimeScript RT kit (Takara, Dalian, China) was used to reverse transcribe mRNAs, while the One Step PrimeScript miRNA cDNA Synthesis Kit (Takara, Dalian, China) was used for miRNAs. Quantification of mRNA and miRNA was conducted with SYBR Green Master Mix (Takara, Dalian, China). QPCR was carried out using the AriaMx Real-Time PCR system (Agilent Technologies, USA). The relative expression levels of mRNA and miRNA were determined using the ΔΔCt method. The miRNA expression levels were normalized to U6 small nuclear RNA (U6-snRNA), while mRNA expression levels were normalized to Beta-Actin (β-Actin). The primer sequences (5′-3′) utilized are as follows: h-actin-F: ACTGGAACGGTGAAGGTGAC, h-actin-R: GGACTTCCTGTAACAACGCAT, h-TFR1-F: CATTTGTGAGGGATCTGAACC, h-TFR1-R: GTCTGGAAGTAGCACGGAAG, h- CD24-F: CTCCTACCCACGCAGATTTATTC, h- CD24-R: AGAGTGAGACCACGAAGAGAC, h-U6-F: CTCGCTTCGGCAGCACA, h-U6-R: AAAATATGGAACGCTTCACG, miR-10a-5p: TACCCTGTAGATCCGAATTTGTG.

### Western blot

The Western blot was conducted following the protocol available at westernblotprotocol.com. In this study, the following antibodies were utilized: anti-CD24 (Abmart, Cat: PU940443S, 1:2000); anti-STAT3 (ABclonal, Cat: A19566, 1:1000); anti-p-STAT3 (ABclonal, Cat: AP0705, 1:2000); anti-TFR1 (ABclonal, Cat: A21622, 1:1000); and anti-rabbit HRP secondary antibody (Cell Signaling, Cat: 7076S, 1:5000).

### Cell viability assay

The cell viability assay was conducted using the Cell Counting Kit-8 (CCK8, Beyotime, Shanghai, China). After transfection for 24, 48, and 72 hours, cells were plated into 96-well plates at a density of 4000 cells per well. Subsequently, 20 μL of CCK8 was added to each well, avoiding exposure to light. The plates were then incubated at 37°C with 5% CO_2_ for 1.5 hours. Finally, cell viability was assessed by measuring absorbance at 450 nm.

### Apoptosis assay

An apoptosis assay was conducted using flow cytometric analysis. Cells (2 × 10^5^) were seeded into 6-well plates for transfection. After 48 hours of transfection, cells were stained with propidium iodide (PI) and annexin V-FITC using an Annexin V-FITC apoptosis assay kit (Beyotime, Shanghai, China) following the manufacturer’s instructions. Briefly, cells were resuspended in 1× binding buffer at a concentration of 1 × 10^6^ cells/ml. PI and Annexin V-FITC (5 μl) were added to 100 μl of the cell suspension, followed by a 15-minute incubation at room temperature in the dark. The stained cells were diluted with 1× binding buffer and analyzed using a flow cytometer (FACS Calibur BD Flow Cytometer, San Jose, CA, USA). The percentages of Annexin V+/PI− (early apoptosis) and Annexin V+/PI+ (late apoptosis) cells were determined according to the manufacturer’s instructions. Data were presented as the rate of total apoptotic cells, including both early and late apoptosis rates. Apoptosis was quantified using a standardized gating strategy on a BD FACS Calibur flow cytometer. First, viable cells were gated based on forward scatter (FSC-A) versus side scatter (SSC-A) to exclude debris and cell fragments. Single cells were then selected on an FSC-H versus FSC-A plot to remove doublets. Quadrant boundaries (Q1–Q4) for Annexin V–FITC/PI plots were established using the following controls acquired under identical instrument settings:(1) Unstained cells to define baseline fluorescence; (2) PI-only and Annexin V-only single-stained controls for compensation adjustment; and (3) Double-positive apoptotic control to verify correct quadrant positioning. The same gates were subsequently applied to all experimental samples. Fluorescence signals were analyzed using FlowJo software (Tree Star, USA). Cells in Q3 (Annexin V^+^/PI^−^) were considered early apoptotic, and those in Q2 (Annexin V^+^/PI^+^) were late apoptotic.

### Immunofluorescence assay

To conduct a cell immunofluorescence experiment, cells were seeded onto coverslips in a 24-well plate and allowed to grow to the desired confluency. The cells were fixed with 4% paraformaldehyde in PBS for 15 minutes and subsequently permeabilized with 0.1% Triton X-100 in PBS for 10 minutes. Non-specific binding sites were blocked by incubating the cells with 5% BSA in PBS for 60 minutes. The CD24 antibody, diluted in 1% BSA in PBS, was applied to the cells and incubated overnight at 4°C. The cells were washed three times with PBS for 5 minutes each, followed by incubation with goat anti-rabbit Alexa Fluor^®^ 488 (Abcam, Cat: ab150113, 1:200), diluted in 1% BSA in PBS, for 1 hour in the dark. The cells were then washed three additional times with PBS. Mounting was performed by applying a drop of mounting medium with DAPI onto a microscope slide and placing the coverslip with cells facing down. The edges of the coverslip were sealed with nail polish, and the cells were visualized using a fluorescence microscope (Nikon, Tokyo, Japan). CD24 immunofluorescence intensity was quantified using ImageJ software (NIH, Bethesda, MD, USA). Images were split into RGB channels, and the green channel corresponding to CD24 fluorescence was selected for analysis. A fixed threshold was applied (Image > Adjust > Threshold) to segment fluorescent regions, ensuring inclusion of signal-positive areas while excluding background. The mean gray value, area, and integrated density were measured using the “Set Measurements” and “Measure” functions, limited to thresholded regions. The fluorescence intensity was normalized by setting the mean value of the control group to 1, and other groups were expressed as relative fold changes.

### Subcutaneous tumor model in nude mice

Male BALB/c nude mice (6–8 weeks old) were procured from the Guangxi Medical University Animal Experiment Center and housed in controlled conditions (23 ± 2°C, 50 ± 5% humidity, light/dark cycle of 10/14 hours). The facilities were approved by the Ethics Committee of Guangxi Medical University Cancer Hospital and Liuzhou People’s Hospital (No. KY2024-067-01). Control lentivirus, miR-10a-5p overexpression lentivirus, and miR-10a-5p inhibition lentivirus were obtained from GenePharma (Shanghai, China). Hep3B cells were infected with these lentiviruses and transduced cells were selected using puromycin (2.5 μg/mL). To establish Hep3B tumors, lentiviral-infected Hep3B cells were suspended in 100 µL of DMEM medium and subcutaneously injected into the right axilla of mice. Sample size (n = 5 per group) was determined based on previous literature using similar hepatocellular carcinoma xenograft models, which demonstrated sufficient statistical power to detect significant tumor growth differences. Mice were randomly assigned to each treatment group, and investigators performing tumor measurements were blinded to group allocation. Tumor volume and body weight monitoring began three days after cell inoculation and continued at 3-day intervals. The study was terminated on day 16, when tumors in some groups approached approximately 1000 mm³, in accordance with institutional animal welfare guidelines to minimize distress and prevent excessive tumor burden. At the experimental endpoint (day 16), mice were euthanized by intraperitoneal injection of 2% sodium pentobarbital (200 mg/kg), in accordance with the AVMA Guidelines for the Euthanasia of Animals (2020). Death was confirmed by cessation of heartbeat and respiration, followed by bilateral thoracotomy as a secondary physical method. Tumors were excised, weighed, and photographed. Tumor volume was calculated using the formula: V (mm³) = 1/6 π × length (mm) × width² (mm²). Histological examination of tumors was conducted using hematoxylin and eosin (HE) staining, while immunohistochemistry (IHC) was performed on 4 μm paraffin-embedded tumor sections. Immunohistochemistry was performed on 4-μm paraffin-embedded tumor sections using an anti-CD24 rabbit monoclonal antibody (clone EPR19925, Abcam, catalog no. ab202073, 1:800 dilution), incubated overnight at 4°C in a humidified chamber. After three PBS washes, tissues were treated with a non-biotin horseradish peroxidase detection system (ZSGB-BIO, China) and photographed at 200× magnification with an Olympus microscope (Tokyo, Japan).

### Luciferase reporter assay

Using the ENCORI database (http://starbase.sysu.edu.cn/index.php), TFR1 was predicted to be directly regulated by miR-10a-5p. The wild-type (WT) 3’UTR (GCUUCCAUGAGAACAGCAGGGUA) and mutant (MUT) 3’UTR (GCUUCCAGGACUAAAGACAACAA) were cloned into psiCHECK-2 vectors. These vectors, either WT or mutant, were co-transfected with miR-10a-5p mimic or control mimic into cells. Forty-eight hours post-transfection, the luciferase signal in the cells was measured using the Dual-Luciferase reagent (Promega, Madison, WI, USA) with a microplate reader.

### Imaging analysis

Preoperative CT image analysis was conducted by two radiologists, Ningbin Luo and Lijuan Liu, with 15 and 12 years of experience in liver CT imaging, respectively. CT images were obtained using either a 320-detector row CT scanner(Canon Medical Systems, Japan) or a 64-detector row CT scanner (Siemens, Germany) with 120 kVp, 150–300 mA, a 5 mm section thickness, a FOV of 350–400 mm, and a standard reconstruction algorithm. Nonionic contrast medium (300 mg I/mL iopromide) was administered at an injection rate of 3 mL/s for a total dose of 100 mL. For the hepatic arterial and portovenous phases, scanning was begun approximately 25 and 60 s after contrast media injection, respectively. Equilibrium phase images were acquired approximately 180 s after contrast media injection. Both radiologists independently reviewed all CT scans and reached a consensus on discrepant cases through joint discussion to minimize interobserver variability and potential batch effects.12 patients were grouped, based on CT imaging three features that were highly recognized and high reproducibility (26, 27). Tumor margins (7, 26): smooth margin, non-smooth margin with focal extranodular, and non-smooth margin with multinodular. Tumor size (≤5 cm; >5 cm) and intratumoral heterogeneity were the top-ranked MVI predicting findings (27, 28). Intratumoral heterogeneity is defined as HCC heterogeneous density within CT venous phase. Based on the CT imaging analysis, 8 patients were classified into either the CT-MVI (+) group or CT-MVI (-) group. 4 patients were used for verification. Cancerous tissues and matched adjacent non-cancerous tissues (5 cm away from the tumor) were harvested from each patient following surgery and analyzed by high-throughput miR sequencing. Iron levels in cancerous tissues were determined using an Iron Colorimetric Assay Kit (Jiancheng, Nanjing, China). RT-qPCR and western blot analysis of the cancerous tissue were performed following standard procedures. All patients were informed about the clinical study and provided signed informed consent.

### Bioinformatics analysis

For small RNA sequencing, 1 μg of total RNA from each sample was used for library preparation using the VAHTS™ Total RNA-seq (H/M/R) Library Prep Kit for Illumina^®^ (Vazyme, Nanjing, China). The resulting libraries were subjected to quality control using the Agilent 2100 Bioanalyzer (Agilent Technologies, USA). High-quality libraries were sequenced as 51-bp single-end reads on an Illumina NovaSeq 6000 platform (Illumina, San Diego, CA, USA) by Genergy Biotechnology Co. Ltd. (Shanghai, China), generating an average of 15–20 million reads per sample. Raw reads were subjected to quality control and adapter trimming using Skewer (v0.2.2). Clean reads were aligned to the human reference genome (Ensembl GRCh38) using Bowtie (v1.0). miRNA annotation was performed against miRBase (v22), Rfam, and RepBase databases. The distribution of different small RNA species was analyzed based on Rfam annotation. Novel miRNAs were predicted using the miRDeep2 pipeline with default parameters. The overall quality metrics of sequencing included the proportion of clean reads (>90%) and mapping rate to the reference genome (>85%). Across all samples, a total of 1417 known were detected. The differential expression analysis of miRNAs obtained from RNA-sequencing was performed using the DESeq2 R package (version 1.40.2). Raw read counts were normalized to eliminate library size effects, and miRNAs with an adjusted p-value <0.05 and |log_2_(fold change)| ≥1 were defined as differentially expressed. Volcano plots and heatmaps were generated using the ggplot2 and pheatmap packages. Functional enrichment of the predicted target genes of these differentially expressed miRNAs was conducted with TopGO (version 2.52.0). Statistical significance was determined using the Benjamini–Hochberg false discovery rate (FDR) correction for multiple testing. miRNAs with adjusted P<0.05 and |log_2_(fold change)| ≥1 were considered significantly differentially expressed. Principal component analysis (PCA) confirmed minimal residual batch effect after normalization.

### Statistical analysis

All quantitative data are presented as the mean ± standard deviation (SD) from at least three independent experiments. Statistical analysis was performed using SPSS 22.0 software (IBM Corp.). One-way analysis of variance (ANOVA) was conducted, followed by Bonferroni *post hoc* tests. Correlation between RNA-seq and qPCR measurements of miR-10a-5p was assessed in R (version 4.2.2). Pearson correlation coefficients and 95% confidence intervals were calculated using the cor.test() function in the stats package (version 4.2.2) after log-transformation of expression values to improve normality. Data preprocessing and filtering were performed using the tidyverse collection Scatter plots with linear regression lines and confidence bands were generated using ggplot2, and paired tumor/adjacent samples were visualized accordingly. A P-value of less than 0.05 was considered statistically significant.

## Results

### Differential expression and functional impact of miR-10a-5p in hepatoma cells

**Figure 1:**
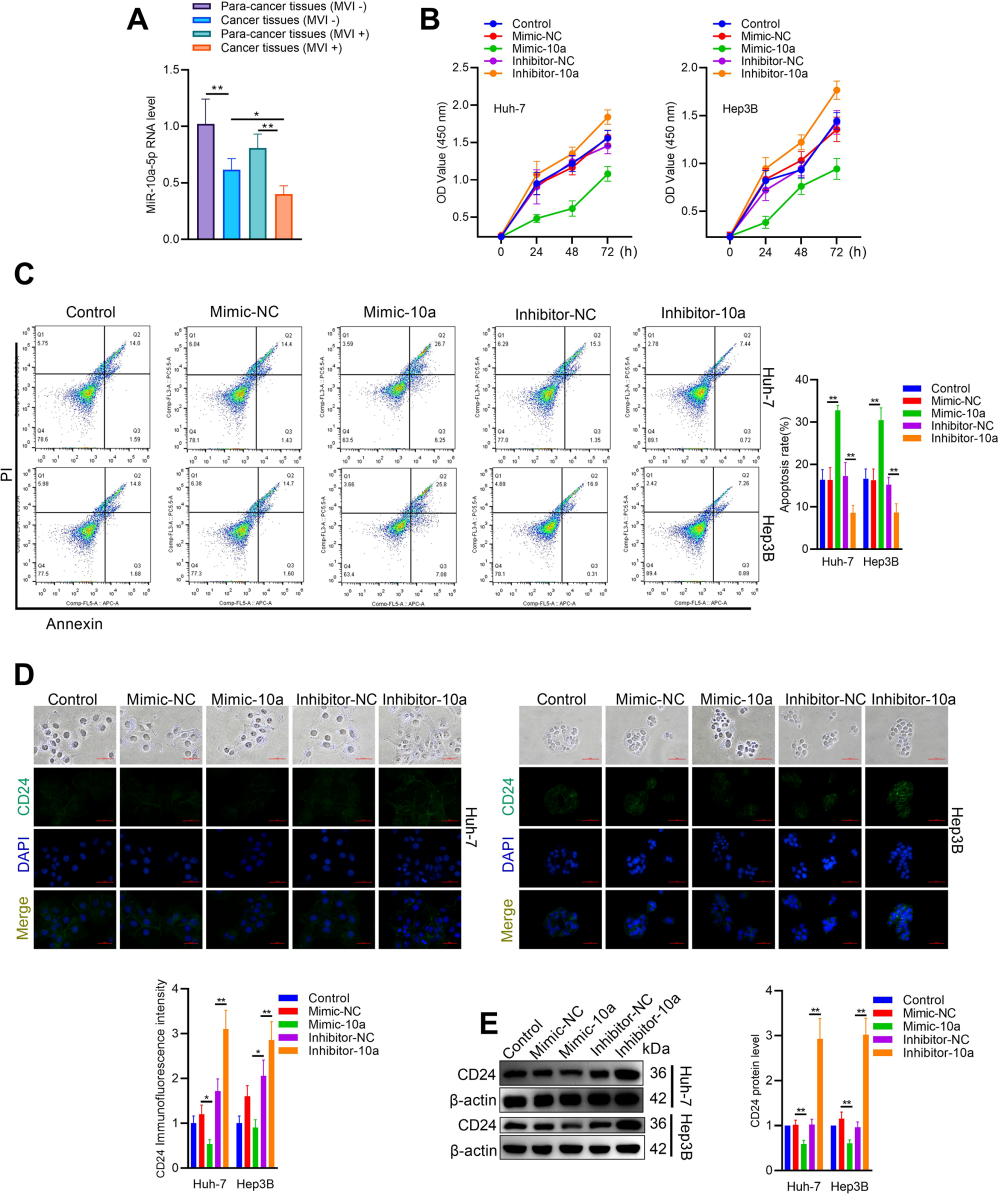
Differential expression and functional impact of miR-10a-5p in hepatoma cells. **(A)** RT-qPCR analysis of miR-10a-5p expression in HCC tissues with or without MVI. **(B)** Cell viability assay (CCK8) of Hep3B and Huh7 cells transfected with miR-10a-5p mimic or inhibitor. **(C)** Flow cytometry analysis of apoptosis in Hep3B and Huh7 cells transfected with miR-10a-5p mimic or inhibitor. **(D)** Immunofluorescence staining of CD24 in Hep3B and Huh7 cells transfected with miR-10a-5p mimic or inhibitor. **(E)** Western blot analysis of CD24 expression in Hep3B and Huh7 cells transfected with miR-10a-5p mimic or inhibitor. The data are presented as the mean ± SD. *P<0.05; **P<0.01.

As illustrated in **Supplementary Figure 3A**, we identified three key imaging features to distinguish between CT-MVI (+) group and CT-MVI (-) group. CT-MVI (+) group HCC CT are characterized by a size exceeding 5 cm, the presence of visible irregular, non-smooth margins and heterogeneous density in venous phase. Conversely, CT-MVI (-) group HCC CT exhibit a smaller profile with size under 5 cm, smooth, well-defined margins indicative of nodular growth patterns, and homogenous density in venous phase. Next, we examined the differential expression profiles of miRNAs between para-tumorous and tumor tissues. Sequencing experiments were conducted on the total RNA extracted from these samples. The sequencing data revealed that miR-10a-5p expression is significantly reduced in liver cancer tissues compared to adjacent non-cancerous tissues (**Supplementary Figure 3B**). RT-qPCR results indicated that miR-10a-5p levels were markedly lower in tumors with CT-MVI compared to those without CT-MVI, suggesting a correlation between miR-10a-5p and CT features (**Figure 1A**). A statistically significant correlation was observed between RNA-seq and qPCR measurements of miR-10a-5p (Pearson R = 0.651, P = 0.0218; R² = 0.424), indicating moderate agreement between the two platforms and supporting the consistency of the observed expression trend. The CCK8 assay showed that the miR-10a-5p mimic decreased cell viability, while the miR-10a-5p inhibitor increased hepatoma cell viability (**Figure 1B**). Additionally, flow cytometry results demonstrated that transfection with the miR-10a-5p mimic enhanced cell apoptosis, whereas transfection with the miR-10a-5p inhibitor reduced apoptosis in hepatoma cells (**Figure 1C**). CD24 functions as a checkpoint inhibitory molecule, enabling macrophage-phagocytosis evasion through its interaction with Siglec 10. We subsequently investigated the effect of miR-10a-5p on CD24 expression. Cellular immunofluorescence and western blot results showed a negative correlation between miR-10a-5p and CD24 expression (**Figures 1D, E**). Together, these findings suggest that miR-10a-5p may act as a tumor suppressor and is associated with CT features.

### *In vivo* tumor suppressive effects of miR-10a-5p

**Figure 2:**
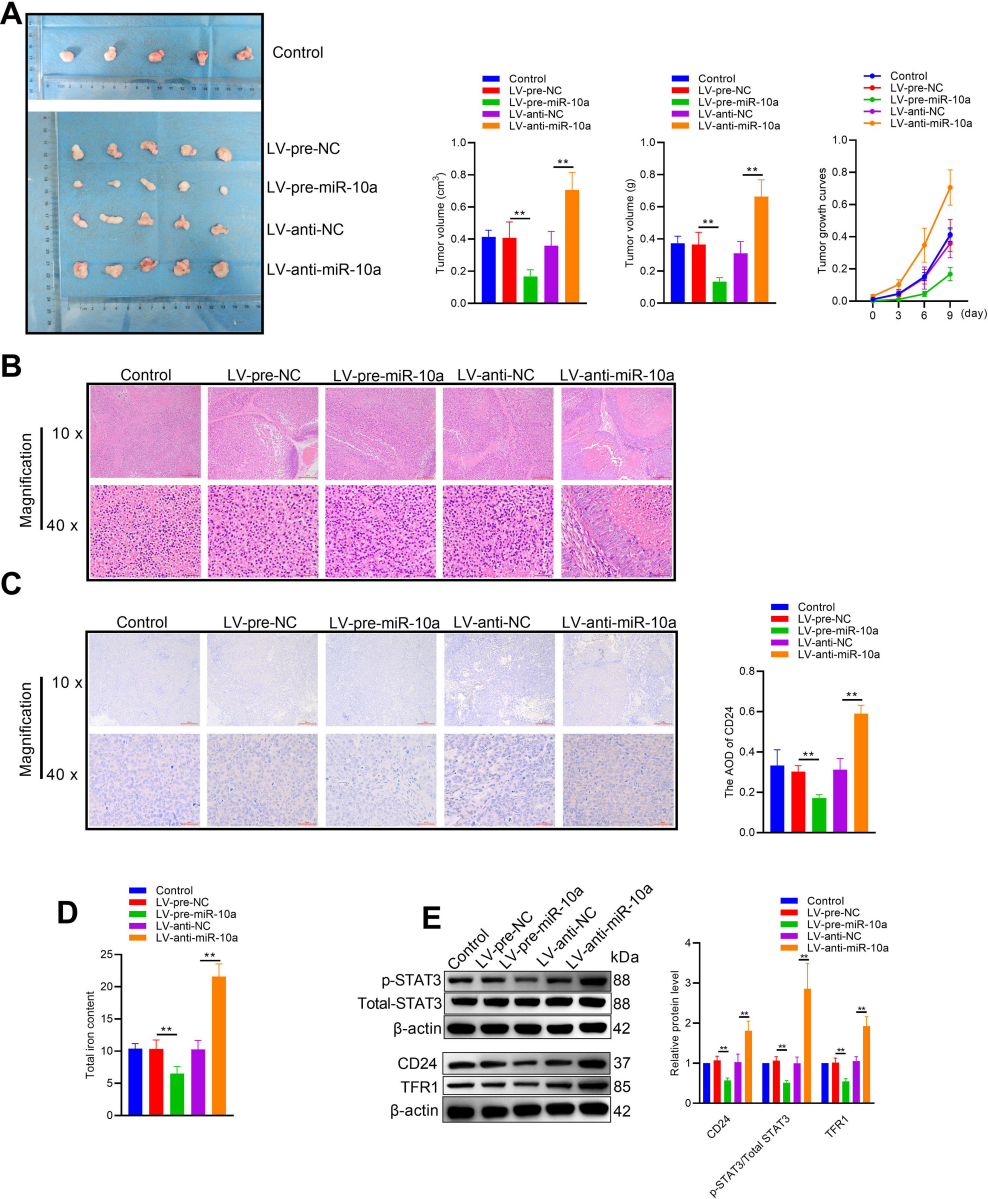
*In Vivo* tumor suppressive effects of miR-10a-5p. **(A)** Tumor growth curves of nude mice subcutaneously injected with Hep3B cells infected with LV-pre-miR-10a, LV-anti-miR-10a, or control lentivirus. **(B)** Hematoxylin and eosin (H&E) staining of tumor tissues from each group. **(C)** Immunohistochemistry staining of CD24 in tumor tissues from each group. **(D)** Total iron content in tumor tissues from each group. **(E)** Western blot analysis of p-STAT3, STAT3, TFR1, and CD24 expression in tumor tissues from each group. The data are presented as the mean ± SD. *P<0.05; **P<0.01.

To determine the *in vivo* tumor suppressive effects of miR-10a-5p, Hep3B cells were infected with either a miR-10a-5p-knockdown lentivirus (LV-anti-miR-10a) or a lentivirus designed to overexpress miR-10a-5p (LV-pre-miR-10a). These modified cells were then implanted subcutaneously into 5-week-old nude mice. Tumors expressing LV-pre-miR-10a grew significantly more slowly than control tumors, while those expressing LV-anti-miR-10a grew more rapidly (**Figure 2A**). Hematoxylin staining confirmed that overexpression of miR-10a-5p led to a marked reduction in tumor growth (**Figure 2B**). Immunohistochemistry of tumor tissues showed lower CD24 protein levels in LV-pre-miR-10a expressing tumors and higher levels in LV-anti-miR-10a expressing tumors (**Figure 2C**). Given that reprogramming of iron metabolism may influence the tumor microenvironment and the immune escape capabilities of tumor cells, we measured the total iron content in the tumor tissues. The results indicated a reduction in total iron levels in the LV-pre-miR-10a group compared to the control group, suggesting that miR-10a-5p is involved in the regulation of iron metabolism (**Figure 2D**). The iron metabolic pathway is regulated by intracellular iron content through interactions between iron-responsive proteins and iron-responsive elements. Previous research has shown that transferrin receptor 1 (TFR1) and STAT3 signaling are closely linked to iron metabolism and tumor immune escape. Consequently, we investigated whether miR-10a-5p regulates TFR1 expression and STAT3 activation. Overexpression of miR-10a-5p was found to downregulate STAT3 phosphorylation, TFR1, and CD24 expression (**Figure 2E**), indicating that miR-10a-5p influences tumor immune escape via the TFR1/STAT3 axis.

### TFR1 as a direct target of miR-10a-5p

**Figure 3:**
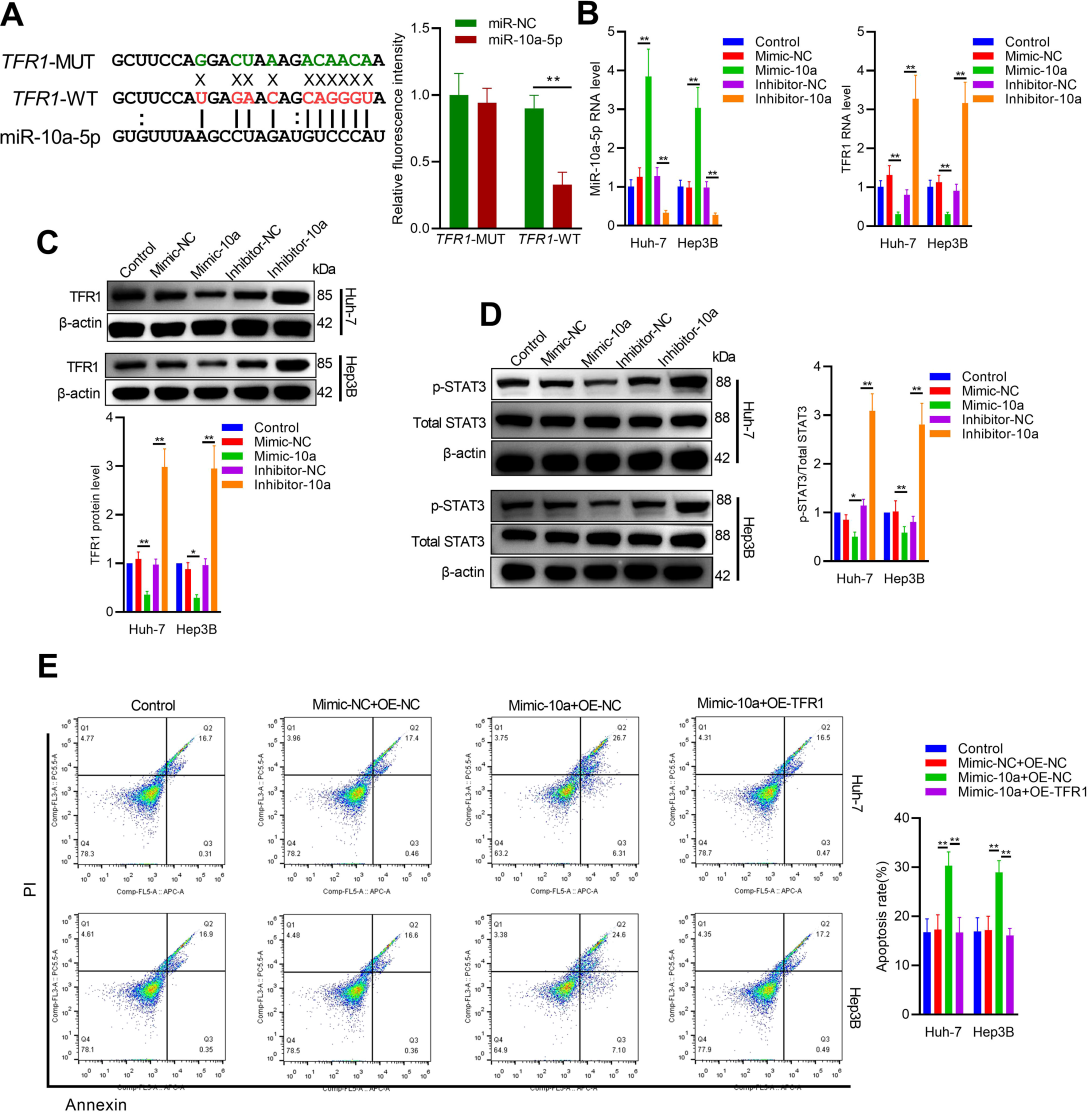
TFR1 as a direct target of miR-10a-5p. **(A)** Predicted binding site of miR-10a-5p in the 3’UTR of TFR1 mRNA. MiR-10a-5p inhibited the luciferase activity of the TFR1-WT luciferase plasmid, while there was no significant difference when the MiR-10a-5p mimic was co-transfected with the MUT luciferase plasmid. **(B)** RT-qPCR analysis of TFR1 mRNA levels in Hep3B and Huh7 cells transfected with miR-10a-5p mimic or inhibitor. **(C)** Western blot analysis of TFR1 expression in Hep3B and Huh7 cells transfected with miR-10a-5p mimic or inhibitor. **(D)** Western blot analysis of p-STAT3 and STAT3 levels in Hep3B and Huh7 cells transfected with miR-10a-5p mimic or inhibitor. **(E)** Flow cytometry analysis of apoptosis in Hep3B and Huh7 cells co-transfected with miR-10a-5p mimic and TFR1 overexpression plasmid. The data are presented as the mean ± SD. *P<0.05; **P<0.01.

**Figure 4:**
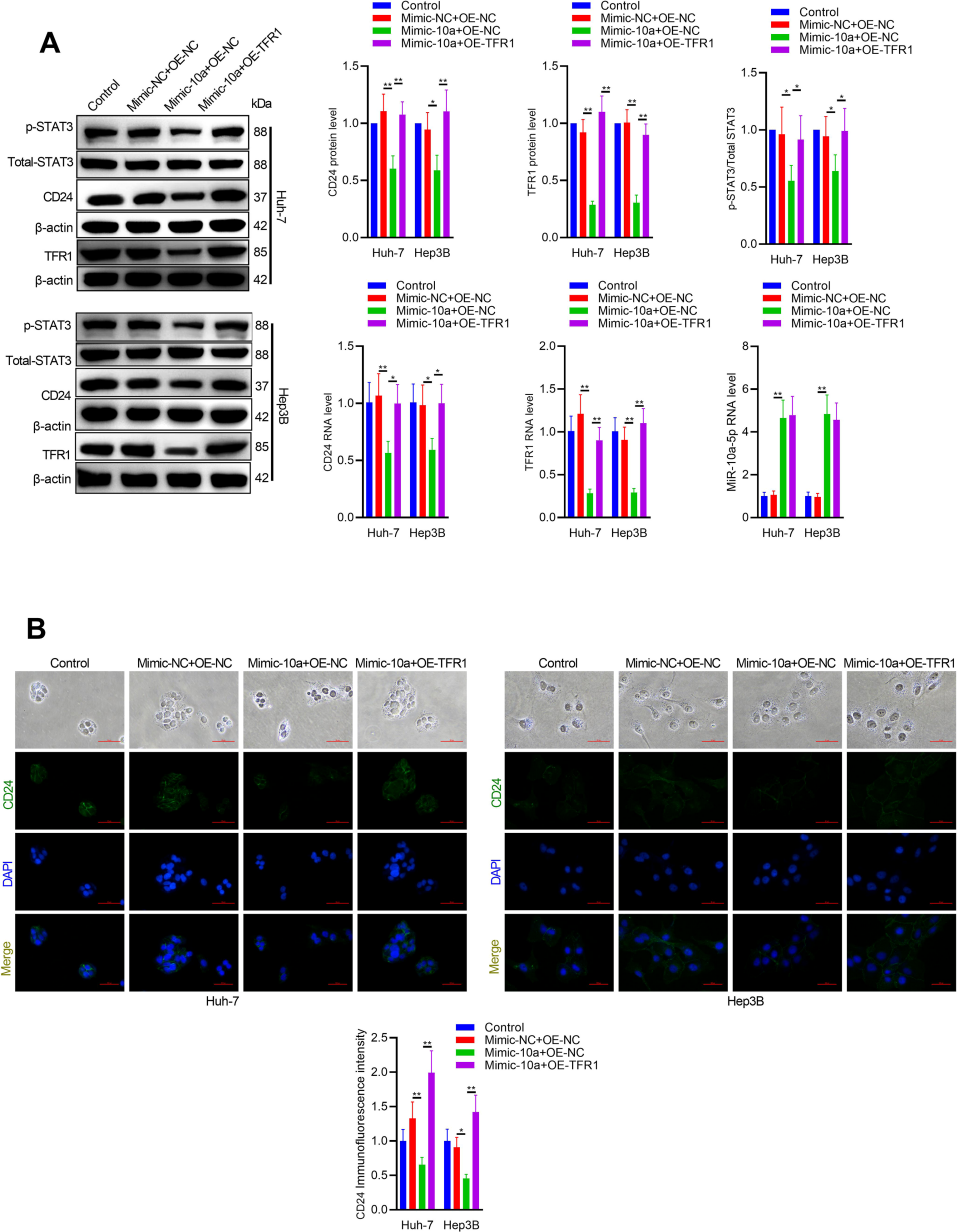
TFR1 overexpression rescues the effects of miR-10a-5p. **(A)** Western blot analysis of CD24, p-STAT3, and STAT3 expression in Hep3B and Huh7 cells co-transfected with miR-10a-5p mimic and TFR1 overexpression plasmid. **(B)** Immunofluorescence staining of CD24 in Hep3B and Huh7 cells co-transfected with miR-10a-5p mimic and TFR1 overexpression plasmid. The data are presented as the mean ± SD. *P<0.05; **P<0.01.

According to ENCORI predictions (http://starbase.sysu.edu.cn/index.php), TFR1 is identified as a direct target of miR-10a-5p (**Figure 3A**). To investigate whether miR-10a-5p exerts its function through TFR1, functional recovery experiments were conducted. As shown in **Figures 3B, C**, miR-10a-5p expression is inversely correlated with the protein and RNA levels of TFR1, indicating miR-10a-5p-mediated regulation of TFR1. Furthermore, overexpression of miR-10a-5p reduced phosphorylated STAT3 levels, whereas miR-10a-5p knockdown increased phosphorylated STAT3 in hepatoma cells, consistent with *in vitro* results (**Figure 3D**). Transfection of miR-10a-5p mimic in hepatoma cells enhanced cell apoptosis, while TFR1 overexpression partially rescued miR-10a-5p-induced apoptosis (**Figure 3E**). Immunofluorescence and western blot analyses further demonstrated that TFR1 overexpression counteracted the effects of miR-10a-5p mimic on CD24 expression and STAT3 activation (**Figures 4A, B**). Additionally, co-transfection experiments with miR-10a-5p inhibitor and TFR1 siRNA confirmed that miR-10a-5p functions by targeting TFR1 (**Supplementary Figures 1**, **2**).

### Biological functions of TFR1 in hepatoma cells

**Figure 5:**
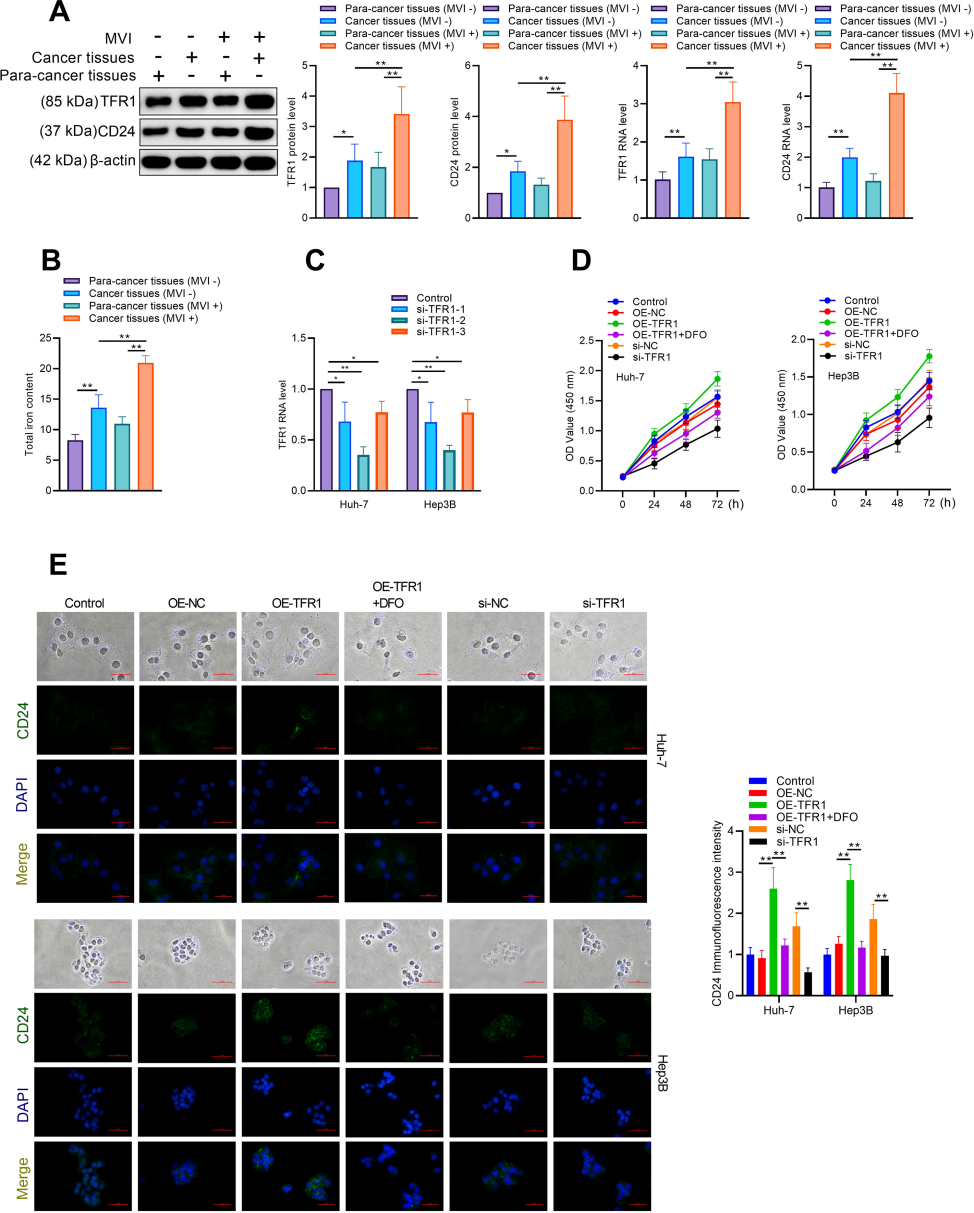
TFR1 is highly expressed in in HCC tissues with MVI. **(A)** Western blot analysis of TFR1 and CD24 in HCC tissues with or without MVI. **(B)** Total iron content in HCC tissues with or without MVI. **(C)** RT-qPCR detection of siRNA targeted TFR1 knockdown efficiency. **(D)** Hep3B and Huh7 cells were transfected with indicated plasmid or siRNA, and then treated with or without Desferrioxamine (DFO). Cell viability was determined by CCK8 assay. **(E)** Hep3B and Huh7 cells were transfected with indicated plasmid or siRNA, and then treated with or without Desferrioxamine (DFO). CD24 expression was determined by immunofluorescence staining. The data are presented as the mean ± SD. *P<0.05; **P<0.01.

**Figure 6:**
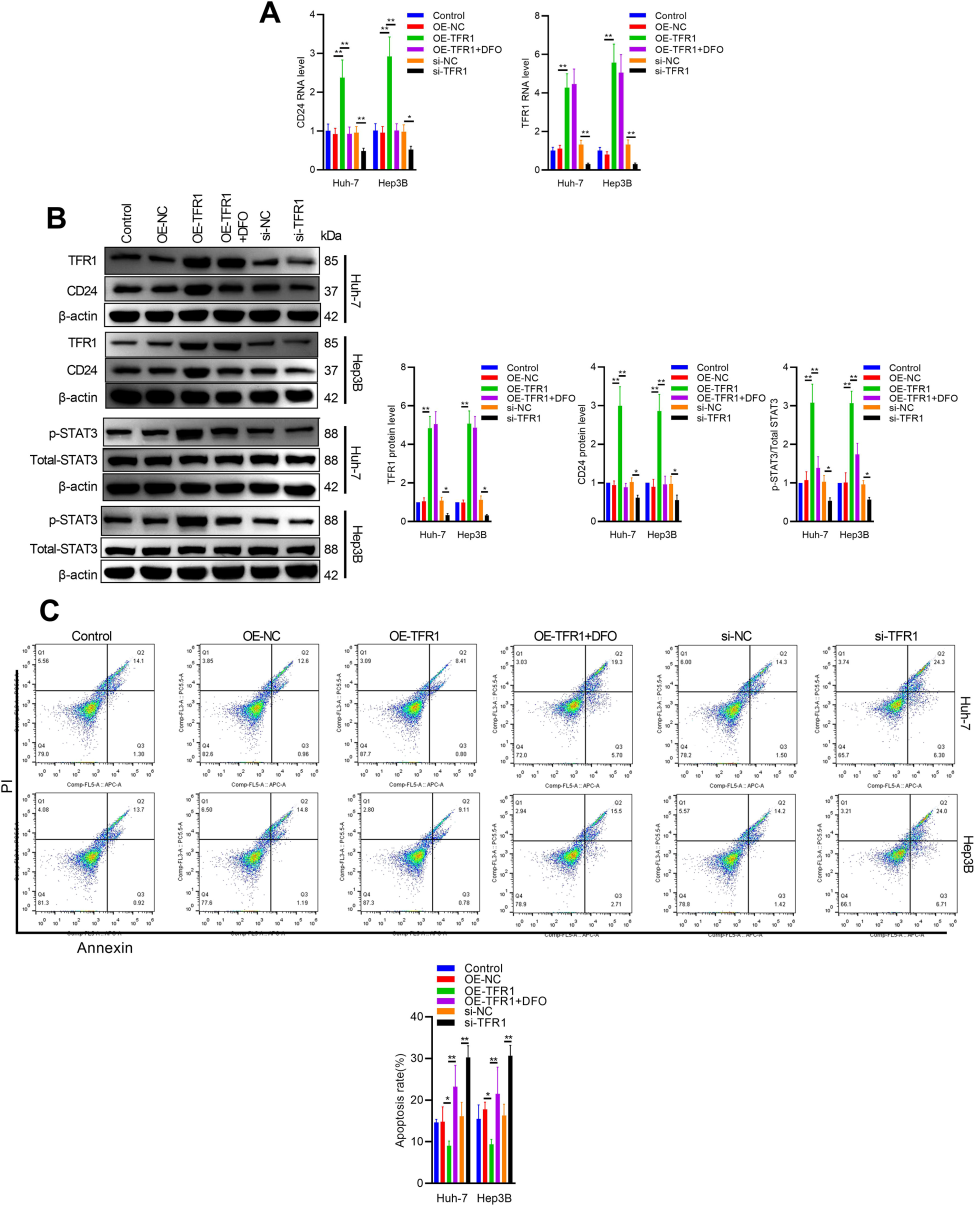
TFR1 regulates STAT3/CD24 signaling in Hep3B and Huh7 cells. Hep3B and Huh7 cells were transfected with indicated plasmid or siRNA, and then treated with or without Desferrioxamine (DFO). **(A)** RNA levels of CD24 and TFR1 were determined by RT-qPCR. **(B)** Protein levels of CD24, p-STAT3, STAT3, and TFR1 expression were determined by western blot. **(C)** Hep3B and Huh7 cells were transfected with indicated plasmid or siRNA, and then treated with or without Desferrioxamine (DFO). Cell apoptosis was determined by flow cytometry analysis. The data are presented as the mean ± SD. *P<0.05; **P<0.01.

TFR1 has been shown to be highly expressed in various cancers to meet the high iron demand of rapidly proliferating cells. To explore the potential biological functions of TFR1 in hepatoma cells, we examined its association with CT features in HCC tumors. Compared to para-cancerous tissues, TFR1 was highly expressed in HCC tissues. Additionally, TFR1 expression was significantly higher in tumors with CT-MVI than in those without CT-MVI, mirroring the expression pattern of CD24 (**Figure 5A**). This suggests that TFR1 is associated with CT-MVI and immune escape in tumors. Elevated iron uptake and hepatic iron overload were also observed in HCC tissues (**Figure 5B**). To further investigate the role of TFR1 in hepatoma cells, we conducted overexpression and knockdown experiments. We designed and synthesized three different siRNAs (si-TFR-1, si-TFR-2, and si-TFR-3) targeting TFR1. RT-qPCR confirmed the specificity of siRNA-mediated knockdown, with si-TFR-2 being the most effective and thus used for subsequent experiments (**Figure 5C**). The CCK8 assay demonstrated that TFR1 knockdown inhibited hepatoma cell viability. Conversely, TFR1 overexpression increased cell viability, an effect that was attenuated by co-treatment with deferoxamine (DFO), indicating that TFR1 promotes hepatoma cell viability by elevating intracellular iron levels (**Figure 5D**). Immunofluorescence staining showed that TFR1 overexpression enhanced CD24 immunofluorescence, which was reduced by DFO treatment (**Figure 5E**). Additionally, TFR1 silencing decreased CD24 expression and STAT3 phosphorylation, while TFR1 overexpression increased CD24 and STAT3 activation, effects that were blocked by DFO treatment (**Figures 6A, B**). TFR1 knockdown protected hepatoma cells from apoptosis, and DFO treatment inhibited TFR1 overexpression-induced apoptosis in hepatoma cells (**Figure 6C**). These results collectively demonstrate that TFR1 overexpression inhibits apoptosis and promotes cell viability through the regulation of cellular iron levels.

## Discussion

This study provides novel insights into the role of miR-10a-5p in hepatocellular carcinoma (HCC) and its association with CT features. Our findings demonstrate that miR-10a-5p acts as a tumor suppressor in HCC and is significantly downregulated in tumors with CT-MVI compared to those without CT-MVI. This downregulation correlates with increased expression of CD24 and TFR1, suggesting a potential mechanism by which miR-10a-5p influences tumor immune escape and iron metabolism. Bioinformatic analysis revealed increased miR-10a-5p levels in human glioma cells, showing a negative correlation with TUSC7, a tumor suppressor candidate (29). In patients with prostate cancer, plasma-derived extracellular vesicles exhibited higher miR-10a-5p expression compared to controls (30). Conversely, ovarian carcinoma samples and cell lines demonstrated decreased miR-10a-5p expression. Upregulating miR-10a-5p in these cells hindered their viability, colony formation, and migratory and invasive capabilities (31). Furthermore, miR-10a-5p acts as an antiviral mechanism in PHEV-induced disease by suppressing Syndecan 1 (32). Our experiments, both *in vitro* and *in vivo*, suggest miR-10a-5p’s tumor-suppressive nature in HCC. When overexpressed, it diminished cell viability, enhanced apoptosis, and curbed tumor growth in xenograft models.

A significant discovery in our research is the opposing relationship between miR-10a-5p and CD24 expression. CD24 functions as a signal preventing macrophage-mediated phagocytosis of cancer cells (14, 33). The fact that miR-10a-5p reduces CD24 expression indicates its potential importance in regulating HCC cells’ immune evasion mechanisms. This becomes particularly significant in MVI cases, where cancer cells in small blood vessels might face increased immune detection risk. HCC can be categorized into five main types: vaguely nodular, single nodular, single nodular with extranodular growth, confluent multinodular, and infiltrative. Research indicates that tumors progressing through these types show increased microvascular invasion and elevated CD24 expression (34).

Through database analysis, our results also reveal a novel regulatory axis involving miR-10a-5p, TFR1, and STAT3 signaling. We identified TFR1 as a direct target of miR-10a-5p, with the microRNA negatively regulating TFR1 expression. The overexpression of TFR1 in HCC tissues, especially in those with MVI, indicates its potential involvement in tumor progression and invasiveness. TFR1 is frequently overexpressed in various cancer types, enabling enhanced iron uptake to meet these metabolic needs (35). In some cancers, TFR1 overexpression has been associated with increased metastatic potential. This may be due to iron-dependent processes that support cell migration and invasion (36). Some studies have suggested that TFR1 may play a role in conferring resistance to certain chemotherapeutic agents (37, 38). Our data showed that TFR1 overexpression to partially rescue the effects of miR-10a-5p on cell apoptosis and CD24 expression underscores the importance of this regulatory pathway in HCC. The link between miR-10a-5p and iron metabolism in HCC is another significant aspect of our findings. The reduced total iron levels in tumors overexpressing miR-10a-5p, coupled with the role of TFR1 in cellular iron uptake, suggests that miR-10a-5p may influence tumor biology by modulating iron homeostasis. This is particularly relevant given the known association between iron overload and HCC risk (39, 40).

The involvement of STAT3 signaling in this regulatory network adds another layer of complexity to the role of miR-10a-5p in HCC. STAT3 is a well-known oncogenic transcription factor involved in various aspects of tumor progression, including cell proliferation, survival, and immune evasion (41, 42). HCC is characterized by abnormally high activity of STAT3. This protein, which acts as a signal transducer and activator of transcription, plays a dual role in its hyperactive state. Research has shown that elevated STAT3 activity contributes significantly to tumor growth and development. Additionally, it aids cancer cells in circumventing the body’s immune defenses, making it a crucial factor in HCC progression (43, 44). Our observation that miR-10a-5p overexpression leads to reduced STAT3 phosphorylation suggests that this microRNA may exert its tumor-suppressive effects, at least in part, through the modulation of STAT3 signaling. These findings have potential clinical implications. The strong association between miR-10a-5p expression and CT-MVI features suggests that CT features could serve as a visualized valuable biomarker for predicting miR-10a-5p expression in HCC patients. This could aid in treatment planning and risk stratification. Furthermore, the tumor-suppressive properties of miR-10a-5p and its regulatory effects on CD24 and TFR1 expression highlight potential therapeutic avenues. Strategies aimed at restoring miR-10a-5p levels or targeting the miR-10a-5p/TFR1/STAT3 axis could potentially enhance anti-tumor immune responses and improve treatment outcomes in HCC patients.

The CT imaging features we identified—tumor size, margin configuration, and intratumoral heterogeneity—showed a strong association with MVI status. These radiologic biomarkers were consistent with our molecular findings, particularly the downregulation of miR-10a-5p in CT-MVI (+) HCC tissues. CT findings characterized by smooth margins, size < 5 cm, and homogeneous venous-phase density may therefore serve as surrogate indicators of favorable molecular profiles, including higher miR-10a-5p expression. Radiogenomics integrates imaging phenotypes with molecular alterations, providing a noninvasive means to infer tumor biology. In HCC, features such as irregular margins, heterogeneous enhancement, and complex texture have been correlated with aggressive gene programs, altered metabolism, and immune dysregulation (45). Consistent with these reports, CT-defined MVI (+) tumors in our cohort frequently exhibited non-smooth boundaries and heterogeneous venous-phase enhancement. Mechanistically, miR-10a-5p downregulation may release inhibition of TFR1, promoting iron uptake and intracellular accumulation. Excess iron triggers oxidative stress and activates STAT3, which upregulates CD24 to facilitate immune evasion via Siglec-10–macrophage signaling. The resulting microenvironmental remodeling—angiogenesis, stromal heterogeneity, and immune suppression—can manifest radiologically as enhancement heterogeneity, indistinct margins, and peritumoral vascular signs. Supporting this, studies reveal that radiomic features in HCC (e.g. texture metrics) correlate with immune and inflammatory pathways and radiogenomic models can stratify tumors by molecular signatures (46). Collectively, these observations suggest that CT features reflecting tumor aggressiveness may serve as imaging surrogates of miR-10a-5p/TFR1/STAT3/CD24 dysregulation. Integrating such imaging–molecular correlations into predictive models could enhance preoperative MVI assessment and guide individualized therapeutic strategies. Furthermore, the integration of imaging and molecular data could facilitate the development of tailored treatment plans. For instance, patients with imaging features suggestive of low miR-10a-5p expression and high risk of MVI might benefit from more aggressive surgical approaches or adjuvant therapies targeting the TFR1/STAT3/CD24 pathway. Conversely, patients with favorable imaging characteristics might be candidates for less invasive treatments or closer monitoring. The non-invasive nature of imaging-based genetic inference is particularly valuable in the context of HCC, where repeated biopsies can be challenging and risky. This approach could enable real-time monitoring of tumor evolution and treatment response, allowing for dynamic adjustment of therapeutic strategies. Moreover, it opens up possibilities for large-scale, population-based studies to further elucidate the relationship between imaging phenotypes and genetic profiles in HCC. It is important to note that while promising, this approach requires further validation in larger, prospective studies. The complex and heterogeneous nature of HCC necessitates robust statistical models that can account for various confounding factors. Additionally, standardization of imaging protocols and analysis methods will be crucial for widespread clinical implementation.

However, several limitations of this study should be acknowledged. While we have demonstrated a clear association between miR-10a-5p and CT features, the exact mechanisms by which this microRNA influences microvascular invasion require further investigation. Additionally, the complex interplay between miR-10a-5p, iron metabolism, and immune escape in the tumor microenvironment warrants more detailed exploration, particularly in the context of different immune cell populations. Although our study revealed a clear correlation between CT features and miR-10a-5p/TFR1/STAT3/CD24 signaling, the limited patient sample size should be acknowledged. Furthermore, the imaging assessment in this study relied on visually evaluated CT features rather than quantitative radiomics analysis. Radiomics-based texture and intensity metrics could provide more objective, reproducible, and fine-grained characterization of tumor heterogeneity. The absence of radiomics therefore represents an important limitation, and future studies with larger cohorts will incorporate quantitative feature extraction to strengthen the imaging–molecular associations. Taken together, these constraints indicate that the present work should be considered a proof-of-concept analysis. Larger, multi-center radiogenomic studies are needed to validate and generalize our preliminary findings.

In conclusion, our study reveals miR-10a-5p as a key regulator of HCC progression and MVI, operating through a novel pathway involving TFR1, STAT3, and CD24. These findings not only enhance our understanding of the molecular mechanisms underlying HCC development and progression but also open up new avenues for diagnostic and therapeutic interventions in this challenging malignancy.

## Data Availability

The data generated or analyzed during the current study are available upon reasonable request from the corresponding authors.
