# Supplementary material for: MiR-10a-5p suppresses hepatocellular carcinoma progression and microvascular invasion by targeting TFR1-STAT3-CD24 signaling axis
Source: Front Oncol. 2026 Jan 2;15:1694441. doi: 10.3389/fonc.2025.1694441 (PMC12807934; doi:10.3389/fonc.2025.1694441)
Supplement: Supplementary file 1 [file DataSheet1.pdf]

Uncropped images(WB)

Western blot antibody validation results.

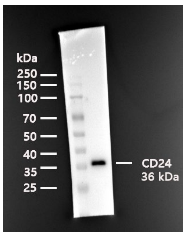	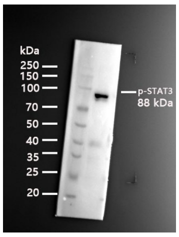	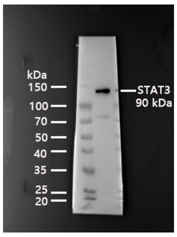	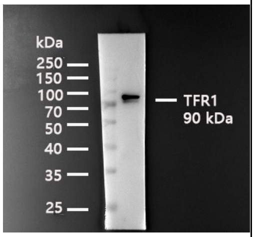	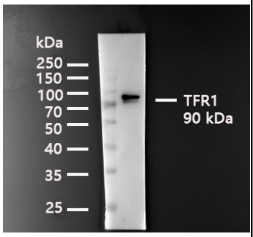
CD24	p-STAT3	STAT3	TFR1	β -actin

Experiment one

<table>
<tr><th colspan="2">HepG2</th><th colspan="2">Huh-7</th></tr>
<tr><td rowspan="2">1--STAT3</td><td>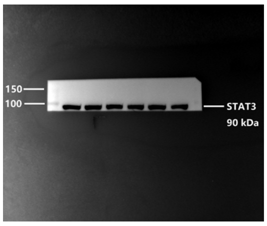STAT3 N1</td><td rowspan="2">1--STAT3</td><td>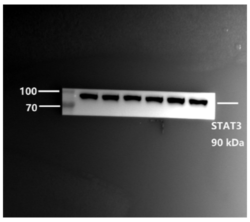STAT3 N1</td></tr>
<tr><td>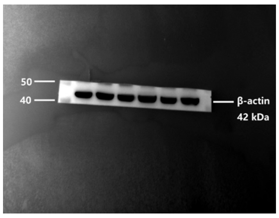β-actin N1</td><td>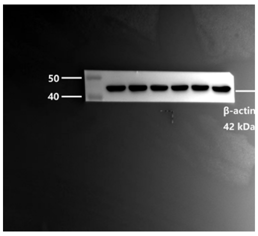β-actin N1</td></tr>
<tr><td>2--p-
STAT3+CD24</td><td>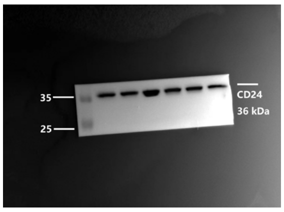CD24 N1</td><td>2--p-
STAT3+CD24</td><td>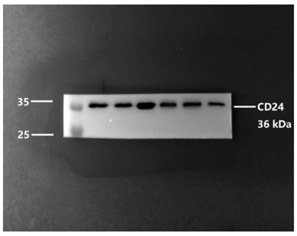CD24 N1</td></tr>
</table>

	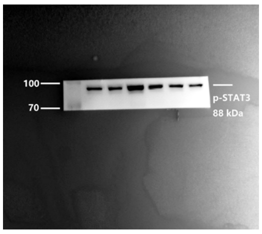<br>p-STAT3 N1		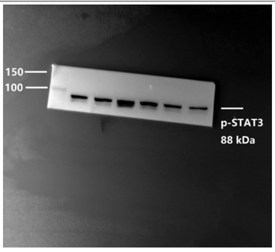<br>p-STAT3 N1
	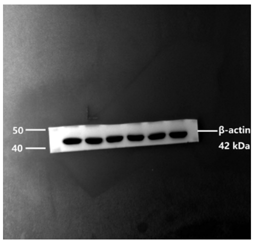<br>β -actin N1		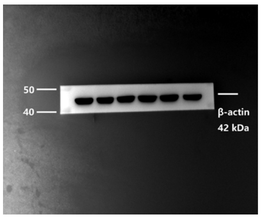<br>β -actin N1
	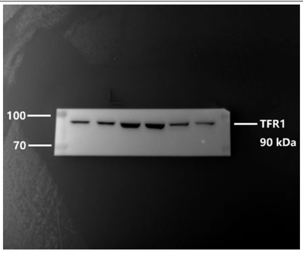<br>TFR1 N1		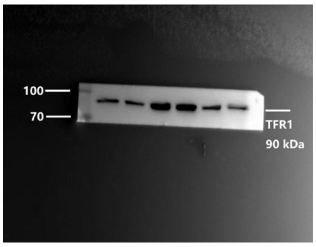<br>TFR1 N1
**3--TFR1**	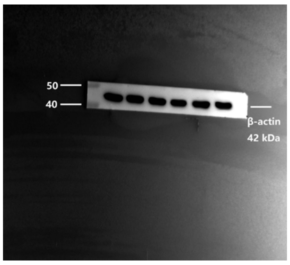<br>β -actin N1	3--TFR1	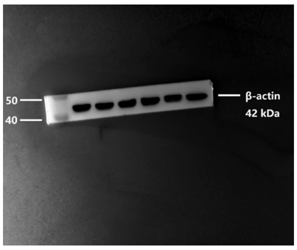<br>β -actin N1

Experiment two

<table>
<tr><th colspan="2">HepG2</th><th colspan="2">Huh-7</th></tr>
<tr><td rowspan="2">1--STAT3</td><td>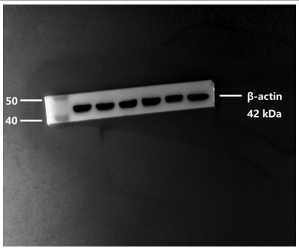

STAT3 N1</td><td rowspan="2">1--STAT3</td><td>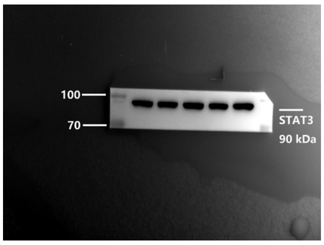

STAT3 N1</td></tr>
<tr><td>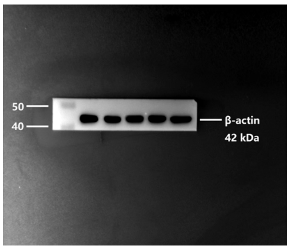

β-actin N1</td><td>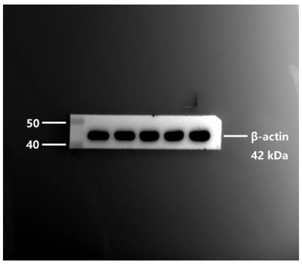

β-actin N1</td></tr>
<tr><td rowspan="3">2--p-STAT3+CD24</td><td>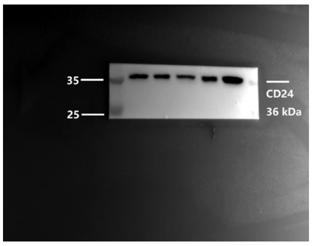

CD24 N1</td><td rowspan="3">2--p-STAT3+CD24</td><td>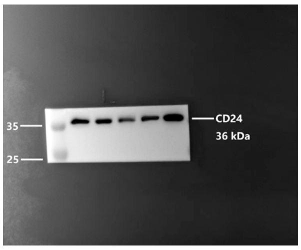

CD24 N1</td></tr>
<tr><td>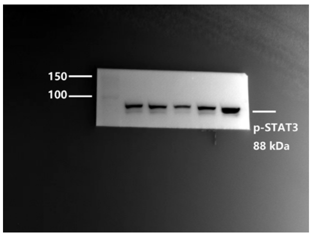

p-STAT3 N1</td><td>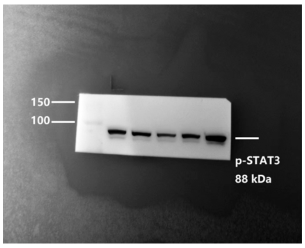

p-STAT3 N1</td></tr>
<tr><td>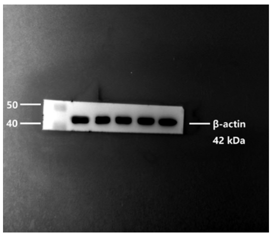

β -actin N1</td><td>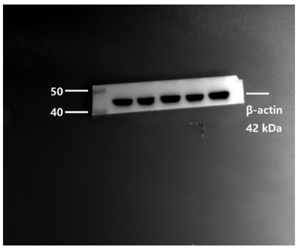

β -actin N1</td></tr>
</table>

<table>
<tr>
<td rowspan="2">3--TFR1</td>
<td>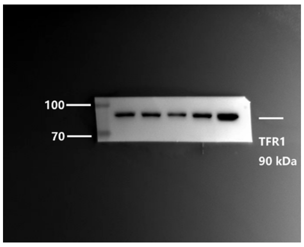

TFR1 N1</td>
<td rowspan="2">3--TFR1</td>
<td>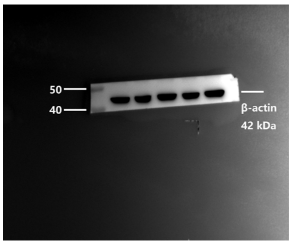

TFR1 N1</td>
</tr>
<tr>
<td>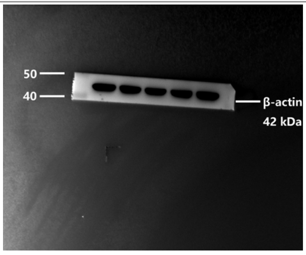

β -actin N1</td>
<td>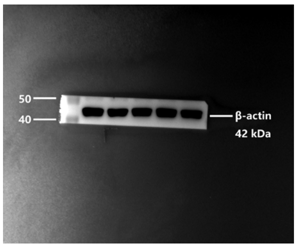

β -actin N1</td>
</tr>
</table>

Experiment three

<table>
<tr><th colspan="2">HepG2</th><th colspan="2">Huh-7</th></tr>
<tr><td rowspan="2">1--STAT3</td><td>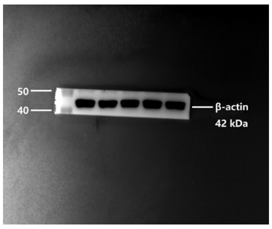

STAT3 N1</td><td rowspan="2">1--STAT3</td><td>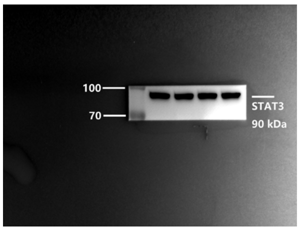

STAT3 N1</td></tr>
<tr><td>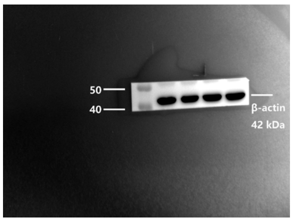

β-actin N1</td><td>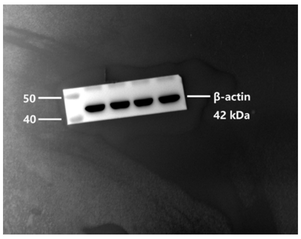

β-actin N1</td></tr>
<tr><td rowspan="3">2--p-STAT3+CD24</td><td>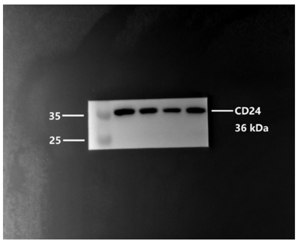

CD24 N1</td><td rowspan="3">2--p-STAT3+CD24</td><td>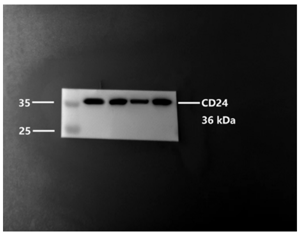

CD24 N1</td></tr>
<tr><td>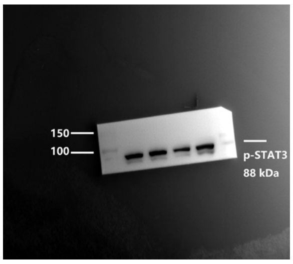

p-STAT3 N1</td><td>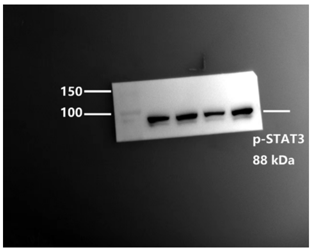

p-STAT3 N1</td></tr>
<tr><td>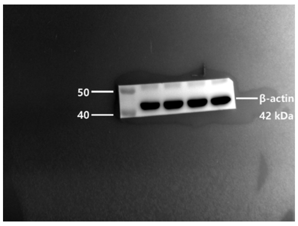

β -actin N1</td><td>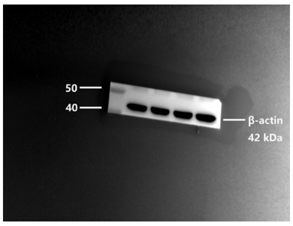

β -actin N1</td></tr>
</table>

<table>
<tr><td rowspan="2">3--TFR1</td><td>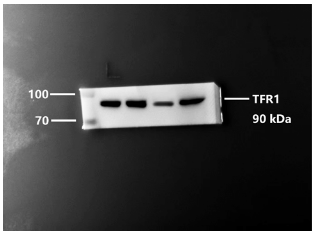
TFR1 N1</td><td rowspan="2">3--TFR1</td><td>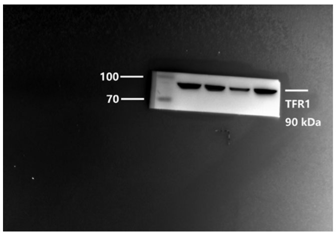
TFR1 N1</td></tr>
<tr><td>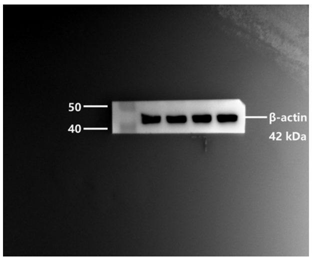
β -actin N1</td><td>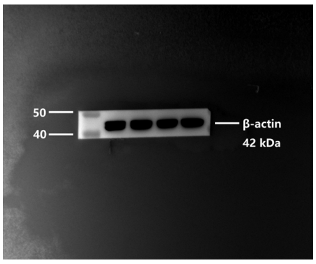
β -actin N1</td></tr>
</table>

Experiment four

<table>
<tr><th colspan="2">HepG2</th><th colspan="2">Huh-7</th></tr>
<tr><td rowspan="2">1--STAT3</td><td>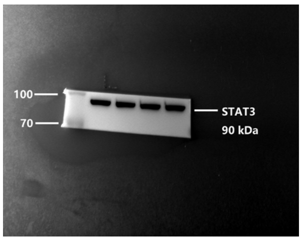

STAT3 N1</td><td rowspan="2">1--STAT3</td><td>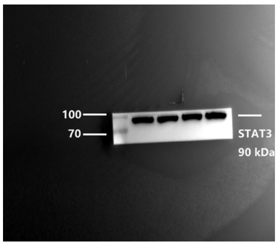

STAT3 N1</td></tr>
<tr><td>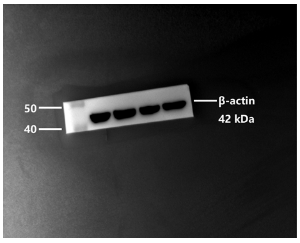

β-actin N1</td><td>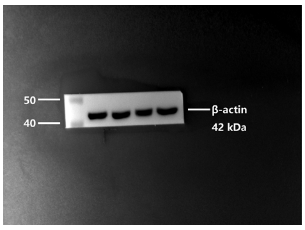

β-actin N1</td></tr>
<tr><td rowspan="3">2--p-STAT3+CD24</td><td>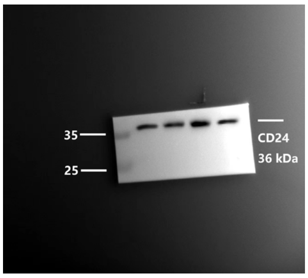

CD24 N1</td><td rowspan="3">2--p-STAT3+CD24</td><td>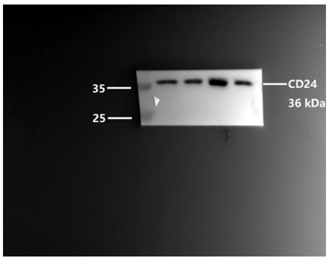

CD24 N1</td></tr>
<tr><td>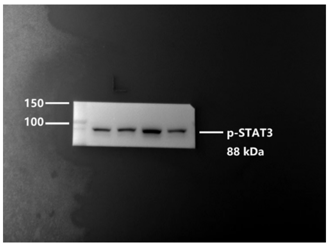

p-STAT3 N1</td><td>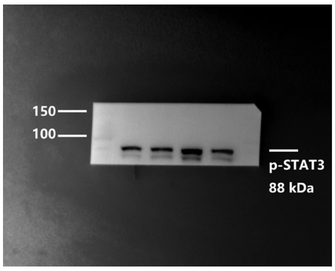

p-STAT3 N1</td></tr>
<tr><td>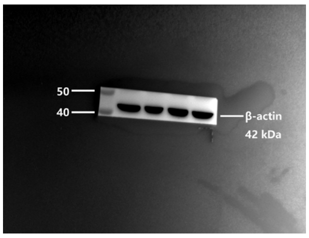

β -actin N1</td><td>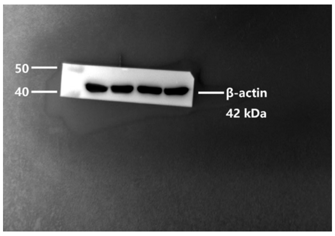

β -actin N1</td></tr>
</table>

<table>
<tr><td rowspan="2">3--TFR1</td><td>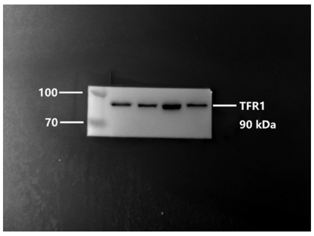

TFR1 N1</td><td rowspan="2">3--TFR1</td><td>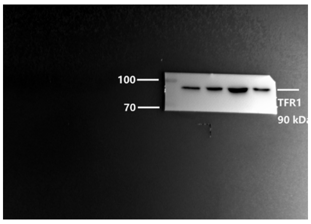

TFR1 N1</td></tr>
<tr><td>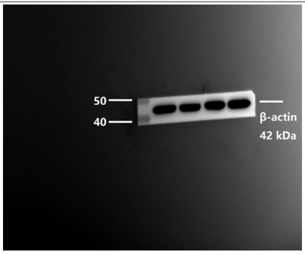

β -actin N1</td><td>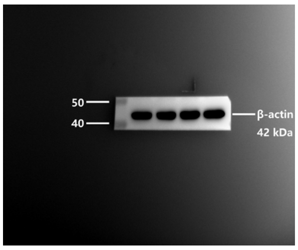

β -actin N1</td></tr>
</table>

Experiment five

<table>
<tr><th colspan="2">HepG2</th></tr>
<tr><td rowspan="2">1--STAT3</td><td>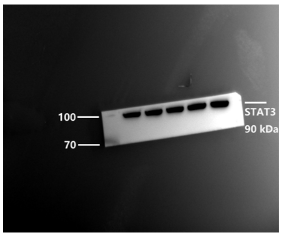

STAT3 N1</td></tr>
<tr><td>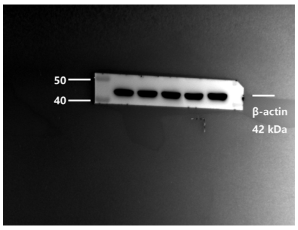

β-actin N1</td></tr>
<tr><td rowspan="3">2--p-STAT3+CD24</td><td>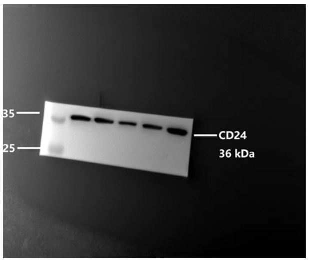

CD24 N1</td></tr>
<tr><td>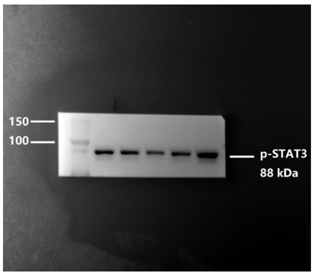

p-STAT3 N1</td></tr>
<tr><td>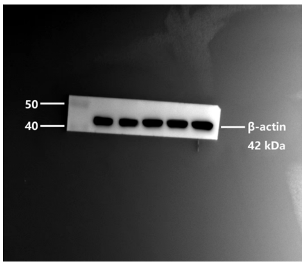

β -actin N1</td></tr>
</table>

<table>
<tr>
<td rowspan="2">3--TFR1</td>
<td>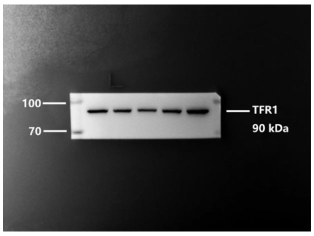

TFR1 N1</td>
</tr>
<tr>
<td>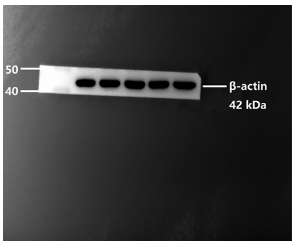

β -actin N1</td>
</tr>
</table>
