# Supplementary figures and images for: MiR-10a-5p suppresses hepatocellular carcinoma progression and microvascular invasion by targeting TFR1-STAT3-CD24 signaling axis
Source: Front Oncol. 2026 Jan 2;15:1694441. doi: 10.3389/fonc.2025.1694441 (PMC12807934; doi:10.3389/fonc.2025.1694441)

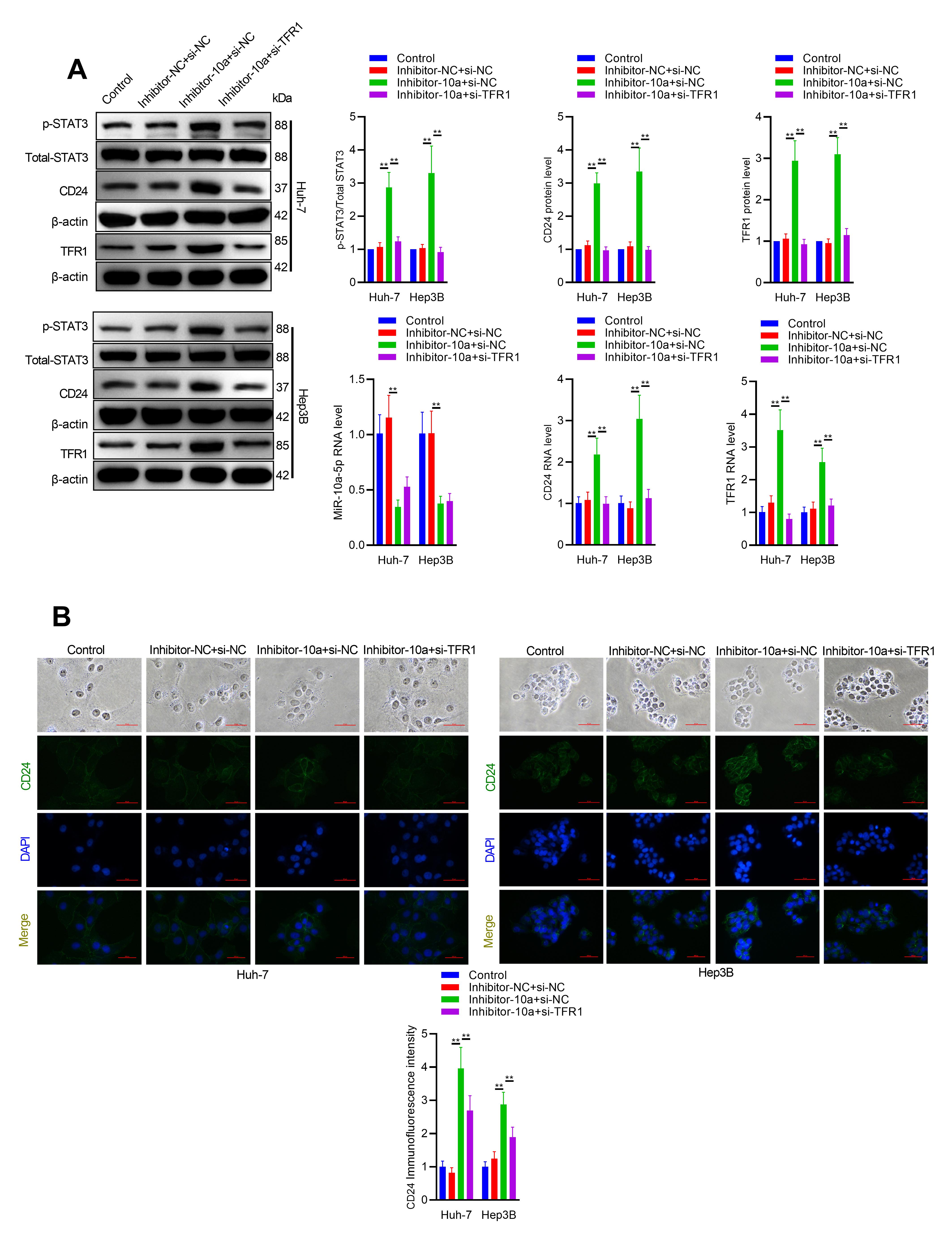

Supplement: Supplementary file 2 [file Image1.jpeg]

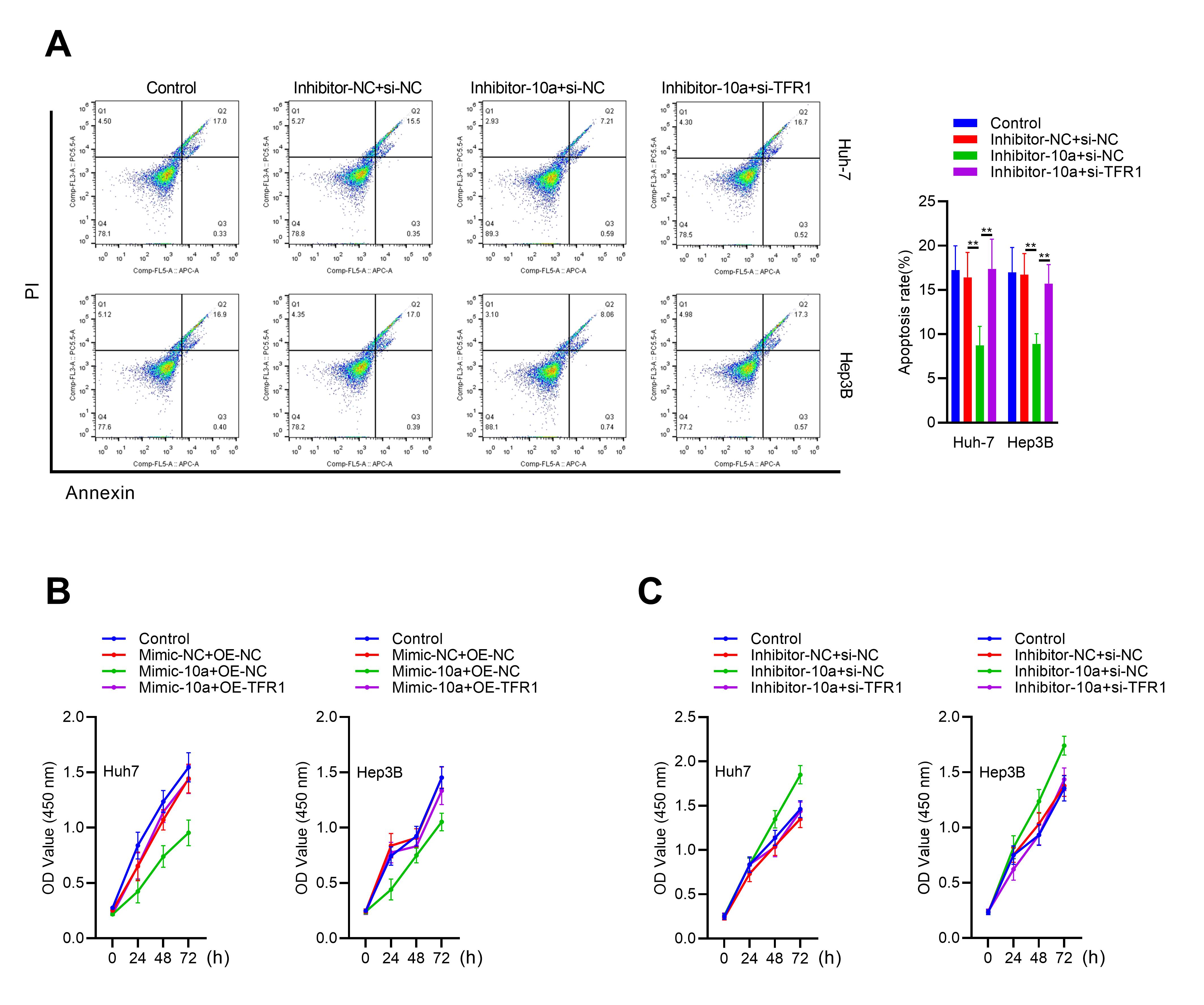

Supplement: Supplementary file 3 [file Image2.jpeg]

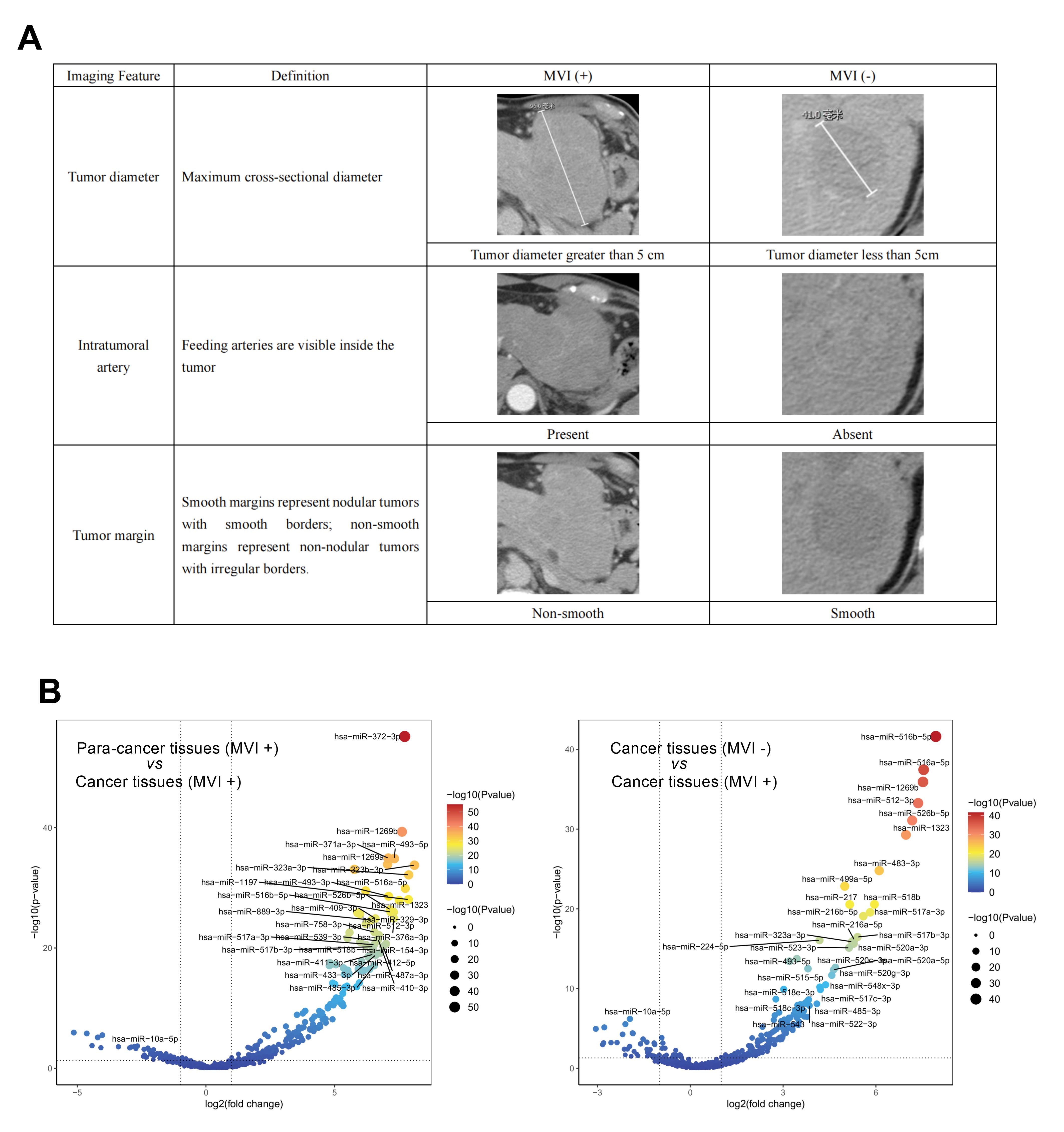

Supplement: Supplementary file 4 [file Image3.jpeg]

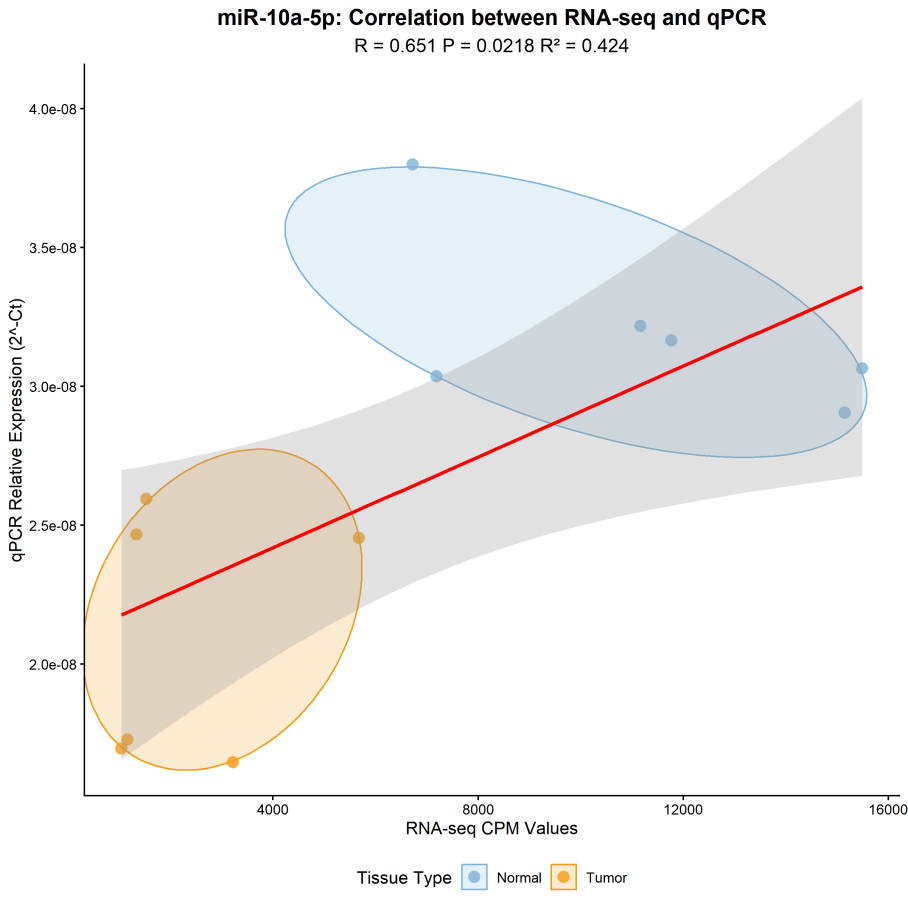

Supplement: Supplementary file 5 [file Image4.tif]
