# Supplementary material for: MiR-10a-5p suppresses hepatocellular carcinoma progression and microvascular invasion by targeting TFR1-STAT3-CD24 signaling axis
Source: Front Oncol. 2026 Jan 2;15:1694441. doi: 10.3389/fonc.2025.1694441 (PMC12807934; doi:10.3389/fonc.2025.1694441)
Supplement: Supplementary file 6 [file Table1.docx]

# Table 1. Baseline Clinical, Pathological, and Imaging Characteristics

| Case | Age (yrs) | Sex | Etiology | Diameter (cm) | AFP (ng/mL) | Child–Pugh | BCLC | Pathologic Grade | Pathology MVI | CT MVI |
| --- | --- | --- | --- | --- | --- | --- | --- | --- | --- | --- |
| 1 | 54 | Male | Without | 10.4 | 126.2 | A | A | II–III | M2 | Positive |
| 2 | 59 | Male | HBV | 3.2 | 59.67 | A | A | I–II | M0 | Negative |
| 3 | 50 | Male | HBV | 6.4 | 1.95 | A | A | II | M0 | Positive |
| 4 | 66 | Male | HBV | 7.5 | >400 | A | A | II–III | M1 | Positive |
| 5 | 52 | Male | HBV | 9.7 | 29.48 | A | A | II | M0 | Positive |
| 6 | 56 | Male | HBV | 1.0 | 379.51 | A | A | III | M2 | Negative |
| 7 | 63 | Male | HBV | 2.0 | 9.42 | A | A | II | M1 | Negative |
| 8 | 66 | Female | HBV | 2.0 | 3.44 | A | A | II | M0 | Negative |
| 9 | 56 | Female | HBV | 2.8 | 48.68 | A | A | II–III | M1 | Negative |
| 10 | 75 | Male | HBV | 2.0 | 8.47 | A | A | II | M0 | Negative |
| 11 | 60 | Male | HBV | 1.0 | >400 | A | A | II–III | M1 | Positive |
| 12 | 57 | Male | HBV | 6.1 | 6.68 | A | A | II–III | M1 | Positive |

Pathological microvascular invasion (MVI) was classified according to the Chinese Guidelines for the Pathological Diagnosis of Primary Liver Cancer:

M0: no MVI detected;

M1: ≤5 MVI foci located within the peritumoral liver tissue (≤1 cm from the tumor border);

M2: >5 MVI foci, or any MVI occurring in liver tissue >1 cm from the tumor margin
